# The role of miRNA and lncRNA in heterotopic ossification pathogenesis

**DOI:** 10.1186/s13287-022-03213-3

**Published:** 2022-12-15

**Authors:** Łukasz Pulik, Bartosz Mierzejewski, Aleksandra Sibilska, Iwona Grabowska, Maria Anna Ciemerych, Paweł Łęgosz, Edyta Brzóska

**Affiliations:** 1 Department of Orthopaedics and Traumatology, Medical University of Warsaw, Lindley 4 St, 02-005 Warsaw, Poland; 2 Department of Cytology, Faculty of Biology, University of Warsaw, Miecznikowa 1 St, 02-096 Warsaw, Poland

**Keywords:** Heterotopic ossification, Noncoding RNAs, microRNA, Long noncoding RNA

## Abstract

Heterotopic ossification (HO) is the formation of bone in non-osseous tissues, such as skeletal muscles. The HO could have a genetic or a non-genetic (acquired) background, that is, it could be caused by musculoskeletal trauma, such as burns, fractures, joint arthroplasty (traumatic HO), or cerebral or spinal insult (neurogenetic HO). HO formation is caused by the differentiation of stem or progenitor cells induced by local or systemic imbalances. The main factors described so far in HO induction are TGFβ1, BMPs, activin A, oncostatin M, substance P, neurotrophin-3, and WNT. In addition, dysregulation of noncoding RNAs, such as microRNA or long noncoding RNA, homeostasis may play an important role in the development of HO. For example, decreased expression of miRNA-630, which is responsible for the endothelial–mesenchymal transition, was observed in HO patients. The reduced level of miRNA-421 in patients with humeral fracture was shown to be associated with overexpression of *BMP2* and a higher rate of HO occurrence. Down-regulation of miRNA-203 increased the expression of runt-related transcription factor 2 (*RUNX2*), a crucial regulator of osteoblast differentiation. Thus, understanding the various functions of noncoding RNAs can reveal potential targets for the prevention or treatment of HO.

## Introduction

### Heterotopic ossification: a brief overview

Heterotopic ossification (HO) is a dysregulation of skeletal muscle homeostasis and regeneration that leads to the formation of mature bone in unusual locations. HO develops in skeletal muscles and surrounding tissues, such as fascia, tendons, skin, and subcutis. Most lesions are small and clinically irrelevant, but extensive HO can limit patient physical functioning and quality of life [1]. The clinical manifestations vary depending on the stage of HO development. Typical clinical symptoms in the early phases are such as localized pain, tenderness, and swelling. At later stages, a limited range of motion (ROM) may affect the joint, resulting in complete ankylosis in the most severe cases [2].

#### Acquired heterotopic ossification

HO formation may occur due to fractures, extensive soft tissue damage, burns, amputations, and combat-related injuries. It can also be triggered by iatrogenic trauma associated with the surgical approach. Up to 90% of acetabulum fractures and 8.6% of distal humerus fractures subjected to surgical treatment result in the formation of HO [3]. It can also occur after arthroscopic procedures and after hip arthroscopy was detected in up to 46% of cases [4]. Approximately 29.9% of patients with total hip arthroplasty (THA) developed HO, but most cases were asymptomatic. However, large lesions occurring in 0.57–2.7% of patients can significantly limit surgery benefits [5]. Extensive HO can influence patient life quality assessed by patient-reported outcome measures (PROMs). It is worth mentioning that mature HO rarely causes pain. However, they substantially reduce the ROM in the affected joint [6]. Patients who were subjected to total hip arthroplasty (THA) with extensive HO lesions (Brooker III, IV) do not benefit from surgery in the ROM aspect, as compared to the preoperative status [7]. HO has been reported to significantly limit joint ROM after revision knee arthroplasty [8], burns [9], and elbow fractures [10]. However, even severe ROM-limiting HOs usually do not cause pain [11].

Another example of acquired HO is neurogenic heterotopic ossification (NHO). The NHO may form around the hip, knee, elbow, and glenohumeral joint due to the response to neuroinflammation signals and systemic changes caused by central nervous system (CNS) injury. Joint mobility problems resulting from NHO often cause nursing challenges [12]. The presence of NHO is associated with a poor functional outcome in patients after CNS injury [13]. In such cases, restricted ROM is the major problem, as pain is often absent due to sensory deficits [14]

Recently, HO has been reported in patients with severe acute respiratory syndrome coronavirus 2 (SARS-CoV-2) infection who require mechanical ventilation. The mechanism of HO in COVID-19 patients is unclear. However, prolonged immobilization, global inflammation, and cytokine storm are likely to be the triggering factors [15, 16].

Zhang et al. described another subtype of HO—heterotopic ossification of the tendon and ligament (HOTL). HOTL includes ossification of the posterior longitudinal ligament of the spine (OPLL) and calcific tendinitis [17]. However, usually OPLL and calcific tendinitis are not mentioned in reviews focusing on HO [1, 18, 19] but are described separately [20, 21]. OPLL can result in radiculopathy or myelopathy that causes spasticity and gait disturbances [17]. It is the most common in the cervical spine. OPLL affects approximately 0.1–4.3% of the world population, with a high prevalence in Asian populations [22]. Unlike other types of acquired HO, it does not result from trauma. The risk factors for OPLL are both genetic, e.g., *COL11A2* [23] and *COL6A1* polymorphisms [24], and environmental, i.e., high-sodium diet [25], obesity [26], and nonalcoholic fatty liver disease [27]. The role of ncRNAs in OPLL is broadly described in a review by Yuan et al. [28]. The calcific tendinitis occurs when repetitive microtrauma acts on the tendons, e.g., in athletes or manual workers. It is common in the rotator cuff tendons [29]. The lesions resemble incomplete ossification and do not contain mature bone but amorphous calcium deposits [30], so they should not be considered a HO subtype.

#### Genetic heterotopic ossification

Heterotopic ossification may be the most notable clinical characteristic of three genetic diseases: fibrodysplasia ossificans progressiva (FOP), progressive osseous heteroplasia (POH), and hereditary Albright osteodystrophy (AHO). Genetic HOs are very severe but rare conditions and belong to the so-called orphan disease family. Osteogenesis induction in FOP is caused by a mutation of the activin A receptor type 1/activin receptor-like kinase 2 gene (*ACVR1/ALK2*; in most cases R206H), which encodes the bone morphogenetic protein (BMP) receptor, type I. POH and AHO are caused by mutations in the *GNAS1* gene, influencing Wingless-related integration site (WNT) and Hedgehog signaling (HH), the key controllers of skeletal maturation and regeneration [31–33]. The prevalence of FOP ranges from 0.4 to 65 cases per 10,000 [34] and the prevalence of AHO is 7.2 cases per million [35]. The epidemiology of POH remains unknown, as less than 60 cases have been reported, so far [36]. Among ossifications of genetic origin, the most severe are those occurring in patients suffering from FOP. FOP is characterized by periodic exacerbations with localized painful soft tissue inflammation that leads to the development of HO in muscles, joints, tendons, and ligaments. Over time, it leads to severe joint limitations and loss of ROM. Most FOP patients are wheelchair-bound in the third decade of life [37]; in addition, they report emotional problems, such as anxiety, depression, or irritability. The severity of pain associated with FOP significantly influences emotional health and overall quality of life [38].

### Possible mechanism of heterotopic ossification

Although many research projects have been devoted to the description of HO, including histological descriptions of the lesions, their progression, and risk factors, the exact molecular processes remain unknown [39]. Patients with HO are most likely to have global changes at the multi-omics level [40], including genetic [41–43], epigenetic [44], transcriptomic, proteomic [45, 46], and metabolic processes [47]. In particular, both genetic HO and acquired HO have already been represented in animal models. The existence of an animal model that satisfies human disease conditions is essential for a detailed explanation of the molecular and cellular mechanisms responsible for disease progression and the preclinical evaluation of potential promising therapeutic tools [31, 39].

Many lines of evidence indicate that the development of HO in skeletal muscle may be the result of pathological differentiation of stem or progenitor cells present in skeletal muscle [39]. However, the identity of these cells is not yet clear. Animal studies suggest that progenitor cells responsible for pathological osteogenesis may differ depending on the HO subtype. Research involving mouse models indicates that endothelial cells, mesenchymal cells, pericytes, tendon, and other connective tissue cells, or circulating stem cells may be the source of HO precursors [48, 49]. In both acquired and genetic HO, the differentiation of precursor cells is initiated by inflammatory cells, including lymphocytes, macrophages, and mast cells [50, 51]. It is accompanied by the release of cytokines and growth factors by immune cells including interleukin 1β (IL-1β), interleukin 6 (IL-6), oncostatin M (OSM), neurotrophin-3 (NT-3), activin A, BMP, transforming growth factor β (TGFβ), and substance P (SP) [50–52]. Differentiation of precursor cells leading to the formation of HO is complemented by increased translation of proteomic biomarkers of HO, such as alkaline phosphatase (ALP), osteocalcin (OCN), osteopontin (OPN/SSP1), and bone sialoprotein (BSP) [45].

The HO can form as any other bone through endochondral or intramembranous ossification. Both processes occur during mammalian skeletal development and bone remodeling during fracture healing [53]. Endochondral ossification occurs in cartilage models of long bones when hypertrophic chondrocytes produce an extracellular matrix that is mineralized in the ossification centers [54]. HO develops through endochondral osteogenesis, which is preceded by infiltration and migration of lymphocytes, fibroproliferation, neovascularity, and cartilage formation [55]. Similarly, in FOP the HO develops with endochondral ossification. Impaired osteochondrogenesis in FOP results not only in extraskeletal bone formation, but also in growth plate dysplasia, early osteoarthritis, and joint deformation [56]. The flare-ups in FOP are accompanied by an elevation of serum cartilage-derived retinoic acid-sensitive protein (CD-RAP), which is a biomarker of chondrogenesis [57]. Opposing to FOP [58] POH ossifications are formed by an intramembranous process in which mesenchymal cells differentiate directly into osteoblasts and form ossification centers [59]. During embryogenesis Intramembranous ossification contributes to the development of the skull, mandible, and middle part of the clavicle [60], and is also responsible for pathological bony bridge formation in growth plate injuries [61].

The precise transduction of signals in HO remains unclear. One of the best-understood regulators of bone development is BMPs, factors from the TGFβ superfamily. BMPs are ligands of transmembrane BMP receptor type I (e.g., ACVR1/ALK2) and type II. From BMP, the signal is transduced with SMAD or non-SMAD pathways. SMADs are cytoplasmic proteins activated by phosphorylation that transmit the signal to the nucleus. The non-SMAD-dependent pathway involves the activation of mitogen-activated protein kinase (MAPK). The signal transmitted by both SMAD and non-SMAD pathways leads to the expression of osteogenesis- and chondrogenesis-promoting transcription factors, such as runt-related transcription factor 2 (*RUNX2*), Osterix (*OSX*) or distal-less homeobox 5 (*DLX5*) [39].

Another mechanism associated with the development of HO that could explain the formation of traumatic HO includes the regulation of the immune response by changes in nuclear factor-κB (*NF-*κ*B*) expression. The NF-κB induces osteogenesis in response to pro-inflammatory ligands of the toll-like receptor (TLR). TLR recognizes damage-associated molecular patterns (DAMPs) released from cells or extracellular matrix after injury, for example, heat shock protein (HSP), high-mobility group box 1 (HMGB1), hyaluronan, or it can be activated by pathogen-associated molecular patterns (PAMPs), for example, lipopolysaccharide (LPS) [62]. Other studies investigated the role of the hypoxic microenvironment in the HO development. Overexpression of hypoxia-inducible factor 1α (*HIF-1α*), a key transcriptional controller of the hypoxic cellular response, may play an important role in pathological bone formation after tissue damage [63]. The rapamycin mammalian target (mTOR) signaling pathway was recently identified to play a role in the pathological osteogenesis [64]. The mTOR is a nutrient sensor and a controller of protein synthesis. The mTOR protein complex-1 (mTORC1) influences cell growth, survival, and proliferation in response to oxygen level, energy status, growth factors, amino acids level [65] or mechanical stimulation [2]. Other factors such as HH and WNT/β-catenin pathways responsible for skeletal maturation and regeneration are also affected by genetic and acquired HO [66]. There is evidence that these pathways can cross talk with BMP signaling, but this network is still being investigated [39, 64, 66].

Recent studies suggest that the development of HO is also regulated by noncoding RNAs. By changing gene expression and mRNA degradation, noncoding RNAs can indirectly stimulate or inhibit the HO formation [45, 67]. Understanding the role of noncoding RNA in HO could lead to highly targeted and efficient HO therapies based on molecules from this group [68].

### Current concepts in HO treatment

Today, pharmacological anti-inflammatory treatment and radiation therapy or both are used to prevent HO. Non-steroidal anti-inflammatory drugs (NSAIDs) are the most frequently administered pharmacological prophylaxis of acquired HO. Recent meta-analysis by Migliorini et al. supports the use of celecoxib, diclofenac, or naproxen in the prevention of HO due to their high effectiveness compared to other NSAIDs [69]. The efficiency of such drugs, both non-selective and selective cyclooxygenase (COX) inhibitors, has been proven in HO prevention. However, their action is not specifically targeted against HO, but rather by inhibiting arachidonic acid prostaglandin production, NSAIDs cause suppression of the inflammatory mechanisms involved in HO [70]. NSAIDs also suppress the transcription factor NF-κB—an immune response regulator that can control osteogenesis [71].

Radiotherapy (RT) is proven to be effective in hip joint surgery [72], non-hip sites such as the elbow or knee [73], and NHO prophylaxis after spinal cord injury [74]. RT is administered before surgery or within 72 h after surgery [75]. According to a meta-analysis by Milakovic et al., there is no difference between postoperative or preoperative RT in preventing HO progression. RT doses higher than 2500 cGy do not result in a better outcome [76]. A single RT dose is less effective than divided to multiple fractions in the prevention of HO after THA [77]. The potential side effects include fatigue, wound healing delay, swollen joints, and very rarely neoplasms that are secondary to RT [78]. Several cases of radiation-induced sarcoma have been reported so far after HO prophylaxis [79–81]. Radiation therapy does not appear to influence implant loosening after THA [82]. There is no consensus, however, as to whether NSAIDs are more effective than RT in HO prevention. A meta-analysis by Pakos et al., focusing on seven randomized trials involving a total of 1143 patients after major hip procedures (THA, acetabular fracture surgery), documented that RT was nearly twice as effective as NSAIDs in HO prophylaxis (Brooker III, IV) [72]. Another meta-analysis by Shapira et al. presented opposing results [83]. Undoubtedly, the cost-effectiveness of HO prophylaxis favors NSAID over RT [84]. The theory that could explain the efficacy of RT in HO prevention is the inhibition of osteogenic differentiation of the MSC. It is accompanied by a decrease in *RUNX2* expression and a decrease in ALP and OCN levels [85].

Experimental approaches directly targeting molecular and signaling processes have been tested in vivo, in animal models, and in humans [86]. Human studies focus on the severe form of HO to FOP. Currently, the most promising effects of these studies in HO inhibition are those regarding the BMP pathway, including receptor ACVR1/ALK2. Another approach is to target NF-κB or mTOR signaling [70].

The clinical trial (LUMINA-1) investigated antibodies against activin A in adult patients with FOP. Garetosmab is a human antibody that binds to activin A, an agonistic ligand of ACVR1/ALK2 [87]. The initial results of the phase 2 study indicate that Garetosmab can reduce the formation of new HO. However, drug administration was halted due to serious fetal adverse events [88, 89]. Recently, a phase 3 trial (OPTIMA) of Garetosmab was registered for adults with FOP [90]. Another monoclonal antibody (DS-6016a) targeted against ACVR1/ALK2 is tested in a phase 1 study in healthy volunteers [91]. Recently orally administered small molecule inhibitors of ACVR1/ALK2 [92] were tested in phase 1 studies on healthy individuals (KER-047 [93], BCX9250 [94]) and phase 2 studies in FOP patients (IPN60130 (FALKON) [95], INCB000928 (PROGRESS) [96]). Dorsomorphin and LDN-193189 are other molecules that inhibit ACVR1/ALK2 but their action is not specific, thus, the potential safety profile of these drugs is questionable [97, 98]. Recent studies in mice have indicated that systemic administration of a neutralizing antibody to activin A inhibits acquired forms of HO also, expanding the therapeutic repertoire of this immunological treatment [99].

Another option for HO drug research is to investigate treatments whose safety profile has already been assessed and which were successful in the therapy of other diseases rather than in new drug development [89]. Anti-leukemic Saracatinib is currently being investigated in adults with FOP (phase 2 trial (STOPFOP)) [100]. It is a potent inhibitor that binds to the ATP pocket of the ACVR1/ALK2 kinase domain and also blocks SMAD phosphorylation and transduction of osteochondrogenic signaling [101, 102]. Another drug repurposed in HO is rapamycin (Sirolimus) which is used to prevent transplant rejection. It influences mTOR signaling that modulates ACVR1/ALK2 and HIF1α action during chondrogenesis in HO [103], effectively blocking HO development in the FOP mouse model [104]. It is currently being investigated in phase 2 in adult FOP patients [105]. Palovarotene, a retinoic acid receptor gamma (RARγ) agonist, previously used in patients with emphysema [106], can inhibit the chondrogenesis phase in HO formation. Palovarotene influences BMP/SMAD-dependent pathway [107] and NF-κB signaling [108] preventing HO formation via endochondral ossification. In a trauma-induced HO rat model. Palovarotene inhibited HO formation and caused down-regulation of chondrogenesis biomarkers, i.e., SRY-box transcription factor 9 (SOX9) and osteogenesis biomarkers (OCN, RUNX2) [109]. It was already tested in adult FOP patients, and a phase 3 clinical trial phase 3 is ongoing (MOVE) [110]. The ongoing clinical trials of FOP drugs are summarized in Table 1.

**Table 1:** Ongoing clinical trials in fibrodysplasia ossificans progressiva

Clinical Trial ID (Acronym)	Study type	Population	Intervention	Comparison	Primary outcome
NCT04818398 (NA) [91]	RCT, Phase 1	Healthy adults (Estimated *n* = 48)	Antibodies against ALK2/ACVR1 (DS-6016a)	Placebo	Safety, tolerability, and pharmacokinetics
NCT05090891 (PROGRESS) [96]	RCT, Phase 2	FOP adults and adolescents (Estimated *n* = 44)	Small molecule inhibitor of ALK2/ACVR1 (INCB000928)	Placebo	Change in HO from BL (WBCT)
NCT05394116 (OPTIMA) [90]	RCT, Phase 3	FOP adults (Estimated *n* = 66)	Garetosmab antibodies against activin A (REGN2477)	Placebo	Change in HO from BL (CT)
NCT03312634 (MOVE) [110]	Open-label, Phase 3	FOP adults, children (Estimated *n* = 110)	Palovarotene—selective RARγ agonist	Untreated FOP subjects from another study	Change in HO (WBCT) compared to untreated subjects from PVO-1A-001, NHS
NCT02279095 (NA) [114]	Phase 2, Open-label, Extension	FOP adults, children (Estimated *n* = 54)	Palovarotene	Different Dosing regimens	Proportion of flare-ups with no new HO (CT or X-Ray), annualized change in new HO (WBCT)
NCT02979769 (NA) [115]	Phase 2, Open-label, Extension	FOP adults, children (Actual enrollment n = 9 participants)	Palovarotene	None, single group Assignment	Annualized change in new HO volume (WBCT)
NCT05027802 (PIVOINE) [116]	Open-label, Rollover Study	FOP adults, children (Estimated n = 87)	Palovarotene	None, single group assignment	All serious and non-serious treatment-emergent adverse events
NCT05039515 (FALKON) [95]	RCT, Phase 2	FOP adults, children (Estimated n = 110)	Selective ALK2/ACVR1 inhibitor (IPN60130)	Placebo	Change in HO (WBCT) and substudy (PET-CT)
NCT04307953 (STOPFOP) [100]	RCT, Phase 2	FOP adults (Estimated n = 20)	Saracatinib Src-kinase inhibitor (AZD0530 Difumarate)	Placebo	Change in HO from BL (WBCT), (PET), patient-reported outcome measures
UMIN 000,028,429 [105]	RCT, Phase 2	FOP adults, children (n = NA)	Rapamycin	Placebo	Physical function at the end of the double-blind stage

Most studies focus on the prevention of HO, and when mature lesions have already formed, the only treatment method is surgical excision [70]. An innovative approach uses osteoclasts, cells responsible for bone remodeling, against mature HO. Osteoclasts were modified with tetracycline, which has a high affinity for bone hydroxyapatite. Artificially engineered osteoclasts with a high affinity for calcified bone capable of HO resorption were successful in treating already formed lesions in tenotomy, intramuscular, and genetic HO mouse models [111].

In recent years, great progress has been made in genetic HO treatment [32, 88, 101]. Recently (January 2022), Health Canada approved palovarotene (Sohonos) as the first drug for Fibrodysplasia Ossificans Progressiva (FOP) [112]. For acquired HO, NSAIDs and RT are still the methods of choice [69]. Emerging evidence indicates the importance of noncoding RNA in the pathogenesis of HO. It suggests that a clinically relevant noncoding RNA signature may be detected in patients with certain risk factors and could be used to predict or prevent HO [113].

### Advances in ncRNA therapy

Noncoding RNAs (ncRNAs) do not encode proteins but play an important role in many biological processes, such as the regulation of gene expression, RNA processing, or protein synthesis. The group of ncRNAs includes, for example, ribosomal RNAs (rRNAs) and transfer RNAs (tRNAs) involved in protein translation, small nucleolar RNAs (snoRNAs) regulating rRNA biogenesis, and small nuclear RNAs (snRNAs) participating in mRNA splicing (broadly reviewed in [117, 118]). Recently, the role of ncRNAs, such as small interference RNA (siRNA), has been intensively investigated in the context of diseases related to the musculoskeletal system [119]. Moreover, ncRNAs have the potential to be applied in the therapy [120]. Here, we focus on two groups of ncRNAs, i.e., microRNAs (miRNAs) and long noncoding RNAs (lncRNAs), which were described to be involved in the formation and progression of HO.

#### microRNA (miRNA)

MicroRNAs are short RNA molecules that consist of approximately 18–30 nucleotides and play an important role in posttranscriptional RNA silencing. Mature miRNA binds to the 3’untranslated region (3’UTR) of its target mRNA, which leads to destabilization or degradation of the mRNA. Regardless of the mechanism of action, the effect of miRNA activity is to prevent protein synthesis coded by target mRNA. Although most of the data underline the inhibitory properties of miRNAs, it should be mentioned that some studies showed that miRNA activity can also lead to up-regulation of gene expression [121–127]. This activation involves argonaute RISC catalytic component 2 gene (*AGO2*) and fragile X mental retardation-related protein 1 gene (*FXR1*) instead of GW182 and was observed in quiescent (*G*_0_) somatic cells and frog oocytes [128, 129]. miR-10a can serve as an example of miRNA-dependent up-regulation of gene expression. Its binding to 5’UTR increased the translation of mRNAs encoding ribosomal proteins during amino acid starvation of mouse embryonic stem cells [130].

**Fig. 1:**
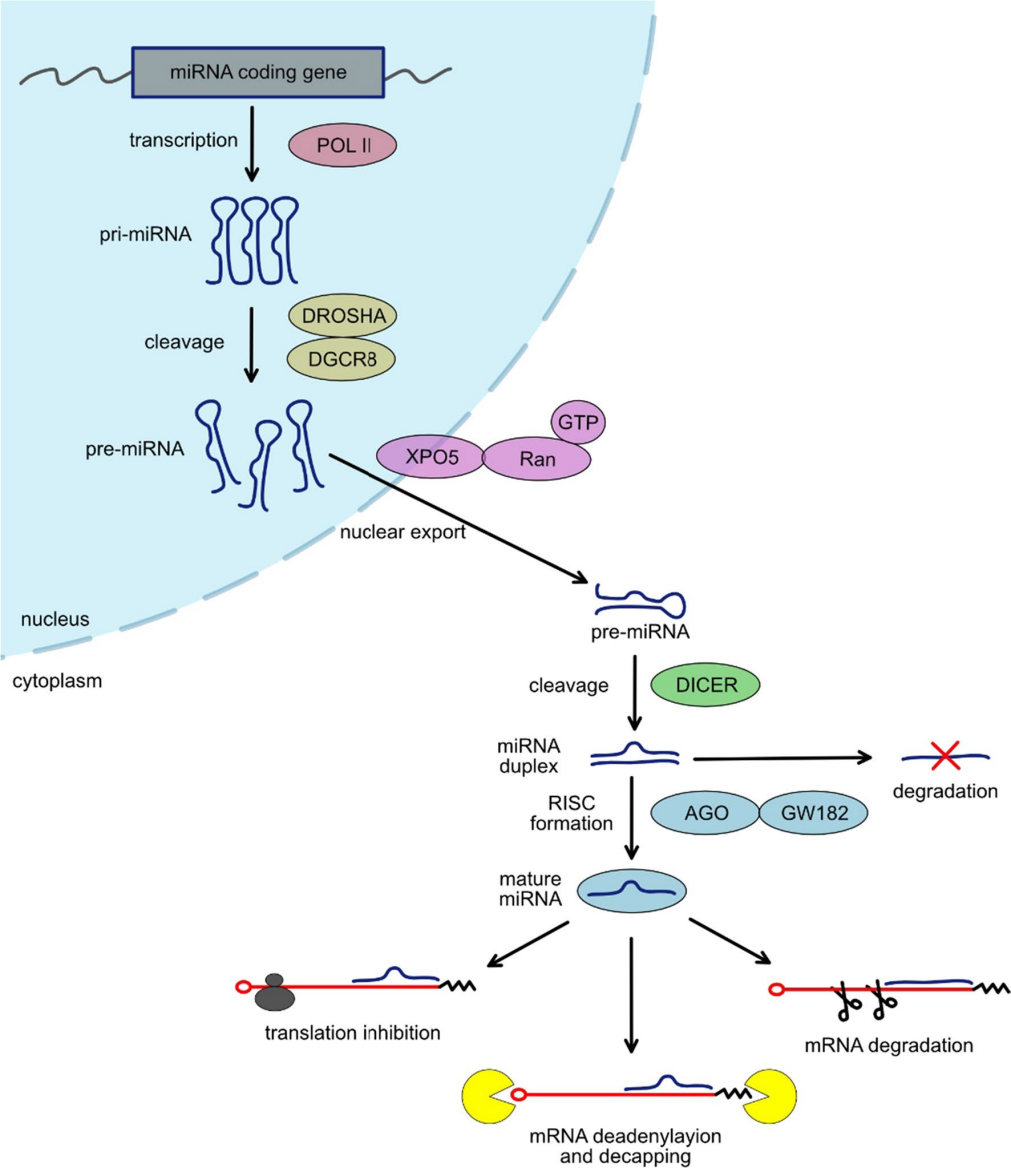
miRNA biogenesis and mechanism of action. miRNA is transcribed by POL II, leading to the generation of often polycistronic pri-miRNA. Pri-miRNA is cleaved by the microprocessor complex which consists of DROSHA and DGCR8 and results in the release of small hairpin-shaped RNA, called pre-miRNA. The pre-miRNA is exported to the cytoplasm by the XPO5/RanGTP complex and cleaved by DICER. DICER cleavage results in the release of a miRNA duplex. One of the miRNA strands becomes degraded, and the other forms a RISC complex with proteins from the AGO and GW182 families. Mature miRNA acts in RISC, and its activity leads to mRNA degradation, mRNA destabilization by deadenylation and decapping, or inhibition of translation. The figure was created for this article; it is not based on any previously published image

The biogenesis of miRNA is quite complex and involves many cellular mechanisms (Fig. 1.). The primary miRNA transcript (pri-miRNA) is a long molecule with a characteristic local hairpin structure within which a mature miRNA sequence is present. Pri-miRNA is cleaved by the endonuclease DROSHA, which, together with the RNA binding protein Di George syndrome critical-related gene 8 (DGCR8), form the microprocessor complex [131–134]. Cropping of the pri-miRNA leads to the formation of pre-miRNA. However, not all miRNA biogenesis involves the Microprocessor complex. Some miRNAs are encoded in so-called mirtrons present in pre-mRNA introns [135–138]. Thus, these pre-miRNAs are generated during pre-mRNA splicing. Furthermore, pre-miRNAs are exported from the nucleus to the cytoplasm by exportin 5 (XPO5)/RanGTP complex [139–141]. In the cytoplasm, pre-miRNA is further processed by other endonuclease DICER, which results in the formation of a miRNA duplex [142–144]. Although both miRNA strands can be functional, during biogenesis one of them is degraded and the other forms an RNA-induced silencing complex (RISC) together with AGO and GW182 proteins [145–149]. After forming the RISC complex, some specific regions of the miRNA structure can be distinguished. Among them, the most important is the “seed” domain, which is directly responsible for recognizing target mRNA. Binding of RISC to mRNA results in mRNA destabilization, mRNA degradation, or inhibition of translation [150]. Furthermore, multiple studies have reported that miRNAs not only act within the cell cytoplasm but also are released into extracellular fluids. These extracellular miRNAs have the potential to be used as biomarkers for diverse conditions [151–153]. Extracellular miRNAs can be delivered to target cells and have the potential to act as autocrine, paracrine, and/or endocrine regulators that modulate cellular activity.

#### Long noncoding RNA (lncRNA)

**Fig. 2:**
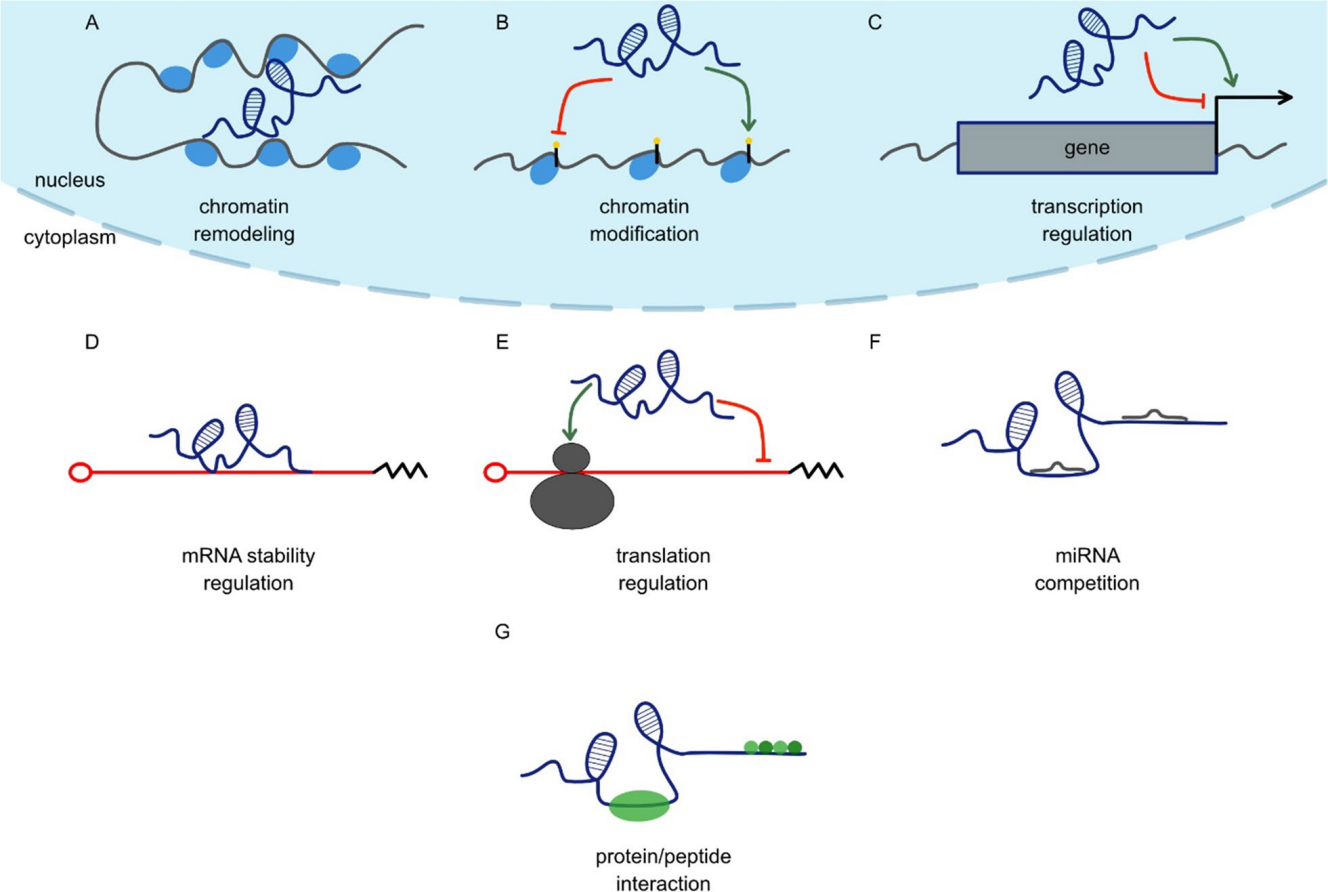
Mechanisms of lncRNA action. Nuclear lncRNAs regulate gene expression by: **A** regulating chromatin remodeling; **B** recruiting chromatin-modifying complexes (such as HDACs); **C** regulating transcription factor activity. Furthermore, cytoplasmic lncRNAs impact mRNA processing through: **D** regulating mRNA stability; **E** regulating translation; **F** acting as competitors for miRNAs; and **G** protein function by binding proteins or peptides. The figure was created for this article; it is not based on any previously published image

Long noncoding RNAs are a very heterogeneous group of ncRNAs, generated via pathways similar to mRNAs and characterized by a minimum size of 200 nucleotides (Fig. 2). In fact, the length of the molecule is the only common feature of all lncRNAs. These transcripts play diverse roles within the cell, acting in both the nucleus and the cytoplasm. However, three main mechanisms of lncRNA activity can be distinguished [117, 118]. In the nucleus, lncRNAs were shown to be involved in the regulation of chromosome structure and mediating chromatin remodeling by recruiting histone-modifying complexes such as histone acetylases or deacetylases (HDACs) (e.g., [154–156]; reviewed in [157]). Furthermore, lncRNAs can directly affect gene expression by binding to enhancer sites or transcription factors [158–160]. lncRNAs acting in the cytoplasm are responsible for posttranscriptional regulation of gene expression by modulating the accessibility or stability of mRNA [117, 118]. Finally, cytoplasmic lncRNAs can directly bind to and inhibit miRNA-dependent gene silencing by functioning as a competing RNA [161, 162]. Therefore, lncRNAs appear to be important regulators of many processes, from the organization of chromosome and chromatin remodeling to the posttranscriptional regulation of gene expression in the cytoplasm.

#### miRNAs and lncRNAs as potential therapeutics

Noncoding RNAs represent very promising tools for the treatment of various diseases [163]. Eleven RNA-based therapeutics have already been approved by the FDA and/or the European Medicines Agency (EMA) [163]. These therapeutics are small interfering RNAs (siRNAs) or antisense oligonucleotides (ASO). They are used, for example, in the therapy of spinal muscular atrophy, Duchene muscular dystrophy, or homozygous familial hypercholesterolemia [163]. Moreover, numerous RNAs are clinically tested. Some of the RNA-based therapies (phase 2 or 3 clinical trials) involve miRNA. However, no lncRNA-based therapy has been available to the clinic, so far [163]. The function of miRNA-based therapeutics relies on restoring or depleting the miRNA or inhibiting their interactions with targets. Few types of miRNA-based molecules are currently being investigated as therapeutics. First, miRNA mimics that have the same sequence as endogenous miRNA and mimic their function. Currently, one molecule, i.e., miR29 mimic, is tested in clinical trials for the treatment of pathological skin fibrosis (NCT02603224, nct03601052). The second type of molecules are antagomiRs that are antisense to specific miRNAs and prevent their interactions with targets. Two such molecules are currently being investigated in clinical trials, these are anti-miR103/107 (NCT020612662 and NCT02826525) and anti-miR122 (NCT01646489, NCT01727934, NCT01872936, and NCT01200420). They are tested in the treatment of type 2 diabetes and hepatitis C virus infection, respectively. Many other miRNA- or lncRNA-based therapeutics are also studied, for example, in the treatment of resistance to cancer therapy [164] and other diseases [165].

#### miRNAs and lncRNAs as potential biomarkers

Several HO biomarkers have been proposed to date, but there is no consensus which of them should be used in clinical practice [166]. Physiological bone turnover indicators based on protein levels are not specific and can change due to other health conditions [167]. Thus, ncRNAs are a promising new group of biomarkers that can be easily isolated from all body fluids and were suggested to be specific markers in many other diseases [168–172]. However, before ncRNA could be widely used as a diagnostic tool, the problem of low expression levels, instability [172], and sequencing costs [208] had to be overcome. Once the mechanism of the complex ncRNA interactions will be uncovered, validation studies and the establishment of cutoff values are needed to enable the application of ncRNA as reliable HO biomarkers. In addition, their specificity as biomarkers should also be investigated in other diseases and reproducible measurement methods should be implemented.

## The role of miRNA and lncRNA in heterotopic ossification

### Dysregulation of the miRNA expression profile in HO

Recently, the role of miRNA and lncRNA has been intensively investigated to understand the background of diseases related to the musculoskeletal system [119] as well as to identify novel drug targets [120]. However, the knowledge about the role of these molecules in the formation of HO is very limited. The role of some of them has been described in the formation of HO resulting from mutations or different types of injury. The study by Ji et al., in which the level of miRNA was compared between patients asymptomatic for HO and those who developed HO, showed that miR205 and miRNA-215 were upregulated and muscle-specific miRNAs, that is, miR1, miR26a, miR133a, miR133b, miR146b, and miR206 were dysregulated [173]. miR205 was described mainly in myoepithelial cells and many cancer types [174] while miR215 was found in osteosarcoma [175]. The other ones which had expression changes were described in this study, i.e., miR1, miR26a, miR133a, miR133b, miR146b, and miR206 belong to muscle-specific miRNAs known as myomiRs. They play an important role in the regeneration of skeletal muscle, including activation, proliferation, and differentiation of muscle stem cells, i.e., satellite cells [176]. Thus, disrupted expression of myomiRs during HO development may be related to defective differentiation of myogenic cells [176].

Comparison of the miRNA profile in the serum of patients with immature HO and mature HO allowed the identification of miR630 as a factor that may be involved in the development of these pathologies [177]. The level of miR630 was significantly lower in the case of both types of HO. Thus, it was suggested that this molecule could serve as an early marker of HO formation [177]. Interestingly, miR630 and its direct target Slug, that is, a member of the Snail family of zinc finger transcription factors, was involved in the endothelial–mesenchymal transition of endothelial cells, which were suggested to be a source of cells responsible for HO formation [177]. Down-regulation of miR630 simultaneously with BMP4 and TGFβ2 treatment increased endothelial cells’ osteogenic differentiation [177].

Another molecule, identified by comparison of miRNAs between the normal bone in patients and primary and mature HO, was miR203 [68]. The level of miR203 decreased in HO; simultaneously, the level of RUNX2 was increased. miR203 directly targets RUNX2 [68]. Furthermore, up-regulation of miR203 inhibited osteogenic differentiation of human osteoblasts. Interestingly, a chemically modified miRNA mimic, named agomiR203, injected into mice that underwent a tenotomy to generate a traumatic HO model, significantly decreased the development of HO, compared to control mice, i.e., injected with phosphate-buffered saline [68].

Next, miR421 is associated with cell proliferation and cancer [178, 179]. However, patients with HO show significantly lower expression of miR421 in their bone and blood, compared to patients who did not develop HO. Thus, miR421 could play a regulatory role in the HO induction [180]. It was documented that BMP2, i.e., the strongest osteogenic induction factor, was the direct target of miR421. Verification of the role of BMP2 in patients with HO induced by humeral fracture showed a significantly increased expression of BMP2 in ossified tissues and blood. Therefore, the development of HO may be related to the up-regulation of BMP2 and down-regulation of miR421 [180].

On the other hand, miR433 is related to numerous diseases. Its expression is increased in fibrotic heart disease rat models [181]. Furthermore, miR433 by targeting the RAP1a and MAPK signaling pathway, miR433 inhibits cancer cell proliferation [182]. Additionally, miR433 plays an important role in esophageal cancer and glioma [183, 184]. Bioinformatics prediction showed that miR433 is a potential upstream regulator of OPN/SPP1 [185]. OPN/SPP1 is a pro-inflammatory factor that affects the adhesion and proliferation of synovial cells [186]. OPN/SPP1 is overexpressed in the cartilage and synovium of osteoarthritis patients with osteoarthritis [187, 188]. The expression of *OPN/SPP1* is regulated, among others, by microRNAs, including miR433. This molecule affects the expression of *OPN/SPP1* by direct binding to the 3′‑UTR of OPN/SPP1 mRNA [189]. In the case of patients with callus and HO in patients with traumatic brain injury (TBI), the expression of *OPN/SPP1* increased significantly and miR433 was reduced, leading to increased accumulation of pro-inflammatory OPN/SPP1 protein in bone tissues and negatively affecting tibial fracture healing of tibial fractures [189].

De Vasconcellos et al. analyzed the miRNA expression profile in samples of patients developing HO and healthy individuals. HO patients demonstrated a unique molecular signature, that is, significantly upregulated miR1, miR26a, miR125b, miR133a, miR133b, and miR206 [113]. When MPCs, i.e., mesenchymal progenitor cells isolated from traumatized muscle tissue of patients, were transfected with miRNA mimics encoding selected miRNAs and induced to undergo osteogenic differentiation, the most potent osteogenic inductors were identified for myomiRs—miR1 and miR206. Next, in vitro and in silico analyses allowed the identification of SOX9, which is involved, among others, in chondrocyte differentiation, as a candidate downstream target of miR1 and miR206 miRNAs in osteogenic differentiation. Investigation of the expression level of *SOX9* in samples from patients developing HO showed that it is downregulated in samples obtained from patients developing patients, as compared to control [113]. Thus, *SOX9* expression is modulated during the development and progression of HO.

It was documented that progenitors, residing in the interstitium of skeletal muscle and expressing the platelet-derived growth factor receptor α (PDGFRα^+^) marker, may participate in the formation of HO [190]. Recently, an in vitro study by Zhu et al. in which human PDGFRα^+^ muscle cells were induced to undergo osteogenic differentiation showed that miR19b-3p could be involved in this process, as its level increases as differentiation progresses. [191]. miR19b-3p acts by indirect induction of OCN, OPN/SPP1, and RUNX2 levels [191]. Furthermore, miR19b3p down-regulates phosphate and tension homolog deleted on chromosome ten (PTEN), which is involved in the regulation of bone formation by inhibiting the phosphoinositide 3-kinase (PI3K)/protein kinase B (AKT) signaling pathway, which was shown during osteogenic differentiation of stem cells [192, 193]. Transfection of PDGFRα^+^ muscle cells with miR19b3p mimic or miR19b3p inhibitor resulted in a decrease in PTEN mRNA when miR19b-3p was overexpressed and increased when miR19b3p was knocked down. Thus, miR19b 3p promotes PDGFRα+ muscle cell osteogenic differentiation of PDGFR+ muscle cells by inhibiting PTEN. Importantly, inhibition of miR19b3p inhibits osteogenesis of PDGFRα+ muscle cells, suggesting that miR9b3p could be a therapeutic target in future therapy against HO. Another molecule regulating the PTEN/PI3K/AKT signaling pathway during osteogenic differentiation of in vitro cultured human primary chondrocytes was miR181a/b-1. Zheng et al. showed that stable lentiviral overexpression of miR181a/b-1 in human chondrocytes enhanced osteogenesis in vitro [194]. Furthermore, PTEN expression was lower in human chondrocytes in which miR181a/b-1 was overexpressed, and consequently, PI3K/AKT signaling was increased [194]. These findings suggest that miR181a/b could also be a target in therapies for bone conditions such as fractures or HO. Another study by Qin et al. investigated miR-17-5p targeting the ankylosis protein homolog (*ANKH*), a gene associated with ankylosing spondylitis (AS). The miR-17-5p inhibitor reduces heterotopic bone formation in samples from the human hip joint capsule [195].

### miRNA that targets ACVR1/ALK2

miR148 was described as a probable target for the development of therapeutic agents against FOP [196]. It directly targeted and downregulated ACVR1/ALK2 mRNA and protein in HeLa cells. Furthermore, in HeLa cells, miR148 downregulated the mRNA of the inhibitor of DNA binding -1, -2, and -3 (ID-1, -2, -3), suppressing the BMP signaling pathway [196]. Point mutations of the *ACVR1/ALK2* gene and their constitutive activation of the BMP signaling pathway are observed in patients with FOP. Constitutively active ACVR1/ALK2 was also shown to cause endothelial–mesenchymal transition of endothelial cells, leading to FOP lesions. However, more studies are needed to examine the role of miR148 in FOP and other HO patients. Another miRNA targeting ACVR1/ALK2 mRNA is miR208a-3p [197]. However, its role in osteoblast differentiation was studied in the mouse hind limb unloading (HLU) model. The overexpression of miR-208a-3p was inhibited, while the silencing of miR-208a-3p with antagomiR-208a-3p promoted osteoblast differentiation [197].

### lncRNA and heterotopic ossification

In vitro studies using human bone marrow mesenchymal stromal cells (hMSCs) showed that lncRNA could also be involved in osteogenesis and bone formation [198]. MSCs were also considered a source of cells responsible for HO formation [39]. The overexpression of lncRNA H19 promoted the osteogenic differentiation of hMSCs in vitro [198]. Furthermore, miR675 encoded by exon 1 of the lncRNA H19 also had an osteogenic effect in hMSC [198]. lncRNA H19 and miR675 downregulated TGFβ1 leading to inhibition of SMAD3 phosphorylation inhibition [198]. On the other hand, TGFβ1 was shown to inhibit osteogenic differentiation of hMSCs. Furthermore, lncRNA H19 and miR675 negatively regulated HDAC4/5, and thus increased the expression of osteoblast markers, such as *RUNX2*, expression [198]. Cells overexpressing lncRNA H19 transplanted subcutaneously more efficiently formed the bone. However, the role of lncRNA H19 or miR675 was not studied in HO patients or mice models of HO formation.

In vitro and in vivo studies using human adipose-derived stem cells (hASCs) showed that lncRNA MIAT (myocardial infarction-associated transcript), which plays an important role in signaling pathways such as Hippo, PI3K/AKT/c-MET, and WNT/β-catenin, is significantly downregulated in osteogenic differentiation of stem cells [199]. *MIAT* knockdown in hASC reverses the inhibition induced by tumor necrosis factor α (TNFα) of osteogenesis. However, its precise role and mechanism of action remained unknown [199]. Another factor that could be involved in HO formation is bromodomain-containing protein 4 (BRD4), that is, a regulator of gene expression involved in osteoclast differentiation. BRD4 belongs to the bromodomain and extraterminal (BET) protein family and participates in the organization of superenhancers and the regulation of oncogene expression. In the HO model, overexpression of BRD4 was associated with an increased level of mitotically associated lncRNA, i.e., Mancr [200], which role was first identified in invasive breast cancer [201]. The BRD4 acting through Mancr lncRNA increased the expression of *RUNX2*, *OSX* gene, and *ALP* encoding gene and, which, as a result, led to HO induction. In this study, a new BRD4-Mancr-RUNX2 pathway was identified, associated with HO signaling, was identified [200]. Nagasawa et al. hypothesized that Mancr activates *RUNX2* expression by another ncRNA—miR-218 [202]. Furthermore, Liu et al. described the use of JQ1, a BRD4-specific antagonist that reduces HO formation [200]. Similarly, silencing Mancr inhibited osteogenesis [200]. Next, Hatzikotoulas et al. hypothesized that variation in the *CAS20* locus that encodes lncRNA is responsible for susceptibility to HO in patients undergoing THA. Upon BMP2 causing osteogenic differentiation of MSCs, the expression of *CASC20* was induced. *CASC20* overexpression was related to RUNX2 and OSX activation and resulted in mineralized tissue formation [42]. The identified studies on ncRNA participating in the formation of HO are listed in Table 2.

**Table 2:** ncRNA participation in the formation of HO

Author (year)	ncRNA	Sample	Setting	HO type	ncRNA expression in HO	Effectors (Direct targets of ncRNA)	Intervention (effect on HO)
Song et al. (2012) [196]	miR-148a	HeLa cells	In vitro	FOP	Decrease	ACVR1/ALK2	NA
Sun et al. (2016) [177]	miR-630	Serum	Humans	Trauma	Decrease	ALP OCN OPN/SSP1 RUNX2 SLUG	miR-630 shRNA ( +) miR-630 mimics (-)
		MVECs	In vitro				
Tu et al. (2016) [68]	miR-203	Bone	Humans Mice	Trauma	Decrease	ALP BSP RUNX2	antagomiR-203 ( +) agomiR-203 (-)
Ju et al. (2019) [180]	miR-421	Serum Bone	Humans	Trauma	Decrease	BMP2	NA
Zhu et al. (2019) [191]	miR-19b-3p	PDGFRα + Muscle cells	In vitro	NA	Increase	ALP OCN OPN/SSP1 PTEN RUNX2	NA
Qin et al. (2019) [195]	miR-17-5p	Joint capsule fibroblasts	Humans In vitro	AS	Increase	ALP ANKH BMP2 COL1Al OCN RUNX2	miR-17-5p mimics ( +) miR-17-5p inhibitor (-)
		AS rat model	Rats				
De Vasconcellos et al. (2020) [113]	miR-1 miR-133a miR-133b miR-206	Muscle MPCs	Humans In vitro	Trauma	Increase	ALP OCN SOX9 RUNX2	NA
Han et al. (2021) [189]	miR-433	Bone Serum	Humans	Trauma, NHO	Decrease	OPN/SSP1	agomiR-433 (-)
Jin et al. (2017) [199]	lncRNA MIAT	hASCs	In vitro	NA	Decrease	ALP OCN RUNX2	sh-MIAT-1 ( +) sh-MIAT-2 ( +) TNF-α (−)
Liu et al. (2021) [200]	lncRNA Mancr	Tendon	Mice	Trauma	Increase	ALP OSX RUNX2	sh-Mancr (−)
		hBMSCs	In vitro	THR Trauma			
Hatzikotoulas et al. (preprint) [42]	lncRNA CASC20	Blood /saliva Bone HMAD hMSCs	Humans In vitro	THR	Increase	OCN OSX RUNX2	NA

## Conclusions

### Role of ncRNA in HO pathogenesis

**Fig. 3:**
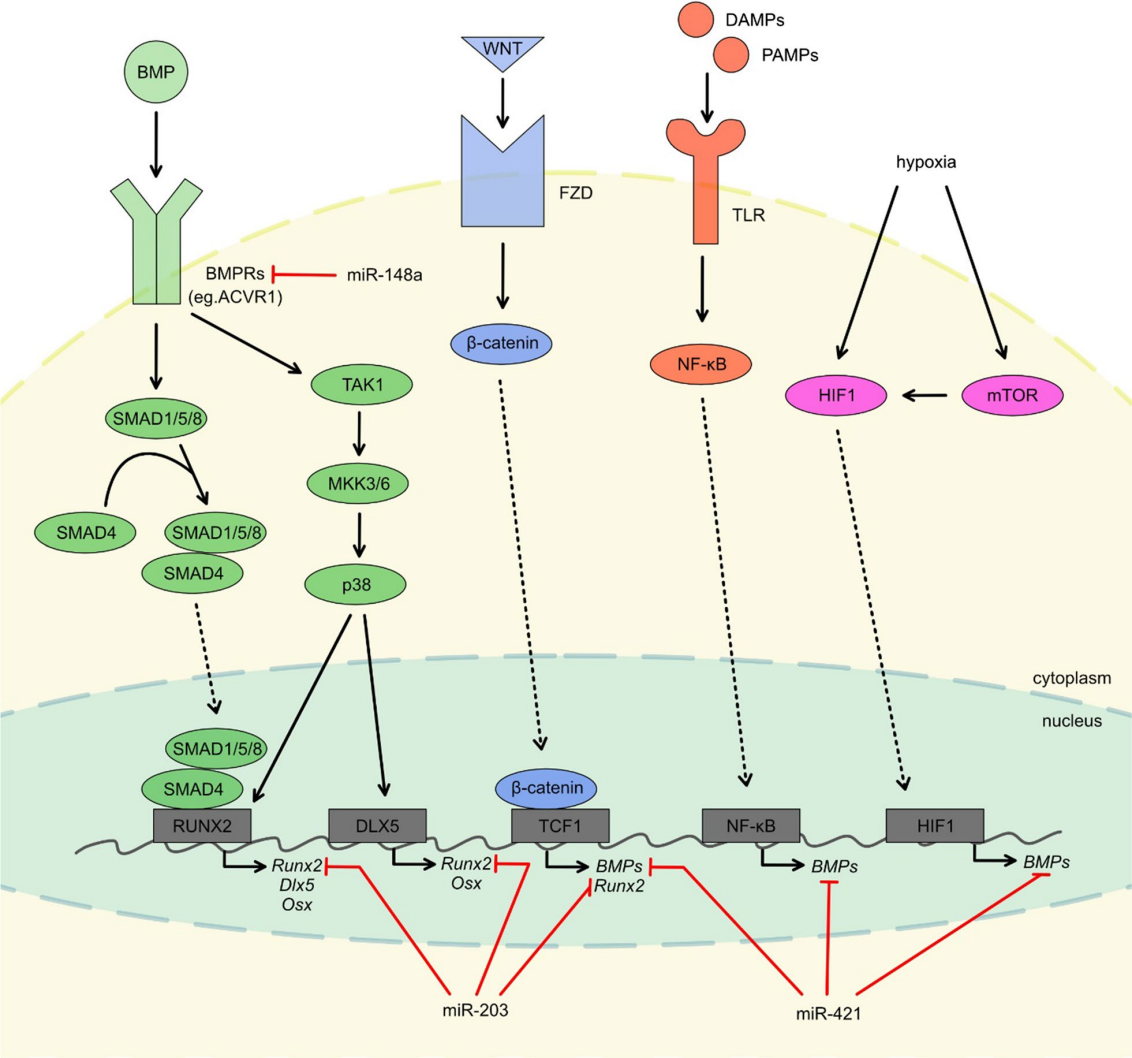
Confirmed miRNA targets possibly involved in HO formation. miR-148a targets ACVR1 coding mRNA that results in a decrease in *ACVR1* expression. ACVR1 is one of the BMPRs that acts as receptors for BMP. BMP binding to BMPRs results in activation of SMAD-dependent or SMAD-independent signaling pathways that leads to activation of transcription factors involved in osteogenesis, such as RUNX2 or DLX5. Other pathways potentially involved in HO formation are WNT, NF-κB or the HIF1 pathway. Activation of all these pathways leads to expression of transcription factors, such as *RUNX2, DLX5,* or *OSX,* which play a crucial role in bone formation, as well as *BMPs*. miR-203 is known to be a negative regulator of *Runx2* translation, while miR-421 acts as a negative regulator of *BMPs* translation. The figure was created for this article; it is not based on any previously published image

The review sums up to date literature on the involvement of ncRNA in the processes that could underlay the HO (Fig. 3). The available data are limited and are based only on several research papers. In some of them, animal models of traumatic heterotopic ossification—Achilles tenotomy in mice [68, 200] or specific muscle/chondrocytes cell lines were used [191]. Other studies included profiling of ncRNA in humans, including post-traumatic HO [113, 173], patients after THA surgery [200] and NHO following CNS injury [189]. Of all published studies, the vast majority concerned only selected miRNA (miR-1, miR-19b-3p, miR-133a, miR-133b, miR-148a, miR-203, miR-206, miR 421, miR 433 and miR-630). Most of the ones that were previously identified by bioinformatic analysis and shown to target specific elements of the HO signaling network elements, e.g., *ACVR1/ALK2* [196], BMP2 [180]. Other miRNA studies aimed at targets such as Slug, which is the regulator of endothelial–mesenchymal transition [177], OPN/SSP1 mRNA [189] or PTEN that controls osteogenic differentiation of muscle cells [90]. Only a few studies analyzed the role of lncRNA in the pathogenesis of HO (lncRNA MIAT, lncRNA Mancr, and lncRNA CASC20) [42, 199, 200]. Two studies proposed a different approach and described complex miRNA profiling and changes in trauma-induced HO [68, 113]. Importantly, it was shown which ncRNA could potentially serve as a biomarker of HO development and its severity. What is important, none of the specific ncRNA that was found to be significantly dysregulated in OPLL (miR-10a, miR-563, miR-199b, miR-182, miR-615, miR-132, lncR MALAT1, and lncR XIST) was investigated in other types of HO or its animal model [28].

Most studies evaluated miRNAs and their impact on bone morphogenesis signaling, including canonical BMP [180, 196] or WNT/β-catenin and MAPK pathways [68]. The expression of the osteogenesis master regulator RUNX2 [68, 113, 177, 191] or levels of well-known osteogenic biomarkers, i.e., OCN [68, 113, 177, 191], OPN/SSP1 [177, 189, 191], ALP [68, 113, 177, 191], and BSP [68] or BMP2 [180] were assessed to determine effect of the miRNAs investigated. Similarly, studies in HO identified lncRNA (MIAT, Mancr, and CASC20) that aim for OSX [42, 200] ALP [42, 199, 200], OCN [42, 199], RUNX2 [42, 199, 200] as their effectors. Until now, no ncRNA studies addressed recently described pathways involved in HO, such as HIF-1α, mTOR, or NF-κB [39].

In few identified studies, researchers successfully blocked osteogenesis or HO formation using miRNA mimics or shRNA (e.g., [177]). Other ncRNAs, i.e., siRNA against RUNX2, OSX, or SMAD, were shown to inhibit HO [203–206]. Thus, ncRNA and its intracellular regulations are a potential target for future HO therapy. Although, to date, none of the miRNA molecules are tested in human clinical trials to treat HO [207].

### Future perspectives of ncRNA in HO

The roles of noncoding RNAs in the pathogenesis of HO remain largely unknown and undefined. Therefore, it is not clear would be the impact of such investigation on the progress in developing appropriate therapies. Each of the research works identifies different RNAs which could indicate that the exact regulatory role of ncRNA in HO remains unknown. Possible research directions for the future, providing such critical but still missing information, include the comparison of the expression profile in different types of HO and normal tissues rather than the comparison of binary interactions.

The therapeutic use of ncRNA in HO requires a design of the delivery method to target cells that ensures stability and safety. One of the challenges of ncRNA therapies is the method of delivery, which is handled by the use of viral (adenovirus, lentivirus) or non-viral vectors [208]. The delivery of viral vectors is efficient, but there is a risk of immunogenicity, toxicity, and carcinogenesis [208]. Therefore, constructs, such as locked nucleic acid [209], cholesterol-conjugated miRNA, lipid particles [210], or bacterially derived minicells, could be used [211]. Another issue is the stability of ncRNA, which could be improved by chemical modifications that prevent it from nuclease [208, 212]. To avoid the immune response, chemical modifications or small molecule inhibitors of ncRNA could be used [163]. To avoid off-target effects, ncRNA can be enriched with cell-specific ligands [208]. Compared to other drugs tested in FOP such as antibodies and small molecules, ncRNA has some potential therapeutic benefits as a therapeutic target. The half-life of ncRNA drugs may be very long, which means more patient-friendly doses [213]. In addition, unlike antibodies, the ncRNA can be transferred from cell to cell by the paracrine effect in exosomes, increasing the bioavailability of the drug in the affected tissue [214]. This review has presented that miRNA and lncRNA are intensively studied in the aspect of HO in both animal models and humans. However, given the complexity of this pathological process involving multiple pathways and possible effector cells in different HO types, the knowledge about the role of ncRNAs in the formation of HO is still very limited.

## Data Availability

Not applicable.

## Abbreviations

ACVR1/ALK2: Activin A receptor type 1/activin receptor-like kinase-2
AGO2: Argonaute RISC catalytic component 2
AHO: Albright osteodystrophy
AKT: Protein kinase B
ALP: Alkaline phosphatase
ANKH: Ankylosis protein homolog
ASO: Antisense oligonucleotides
AS: Ankylosing spondylitis
BL: Baseline
BMP: Bone morphogenetic protein
BRD4: Bromodomain-containing protein 4
BSP: Bone sialoprotein
CNS: Central nervous system
CD-RAP: Cartilage-derived retinoic acid-sensitive protein
COX: Cyclooxygenase
DAMP: Damage-associated molecular pattern
DGCR8: Di George syndrome critical-related gene 8
DLX5: Distal-less homeobox 5
EMA: European Medicines Agency
FOP: Fibrodysplasia ossificans progressiva
FXR1: Fragile X mental retardation-related protein 1
hASCs: Human adipose-derived stem cells
HDAC4/5: Histone deacetylase 4/5
HH: Hedgehog
HIF-1α: Hypoxia-inducible factor 1α
HLU: Hindlimb unloading
HMGB1: High-mobility group box 1
hMSCs: Human bone marrow mesenchymal stromal cells
HO: Heterotopic ossification
HOTL: Heterotopic ossification of the tendon and ligament
HSP: Heat shock protein
IL-1β: Interleukin 1β
IL-6: Interleukin 6
lncRNA: Long noncoding RNA
LPS: Lipopolysaccharide
Mancr: Mitotically associated long noncoding RNA
MIAT: Myocardial infarction-associated transcript
miRNA: MicroRNA
MAPK: Mitogen-activated protein kinase
MPCs: Mesenchymal progenitor cells
MVECs: Microvascular endothelial cells
mTOR: Mammalian target of rapamycin
mTORC1: Mammalian target of rapamycin protein complex-1
ncRNA: Noncoding RNA
NF-κB: Nuclear factor-κB
NHO: Neurogenic heterotopic ossification
NSAIDs: Nonsteroidal anti-inflammatory drugs
NT-3: Neurotrophin-3
OCN: Osteocalcin
OPLL: Ossification of posterior longitudinal ligament of the spine
OPN/SSP1: Osteopontin
OSM: Oncostatin M
OSX: Osterix
PAMP: Pathogen-associated molecular pattern
PET: Positron emission tomography
PI3K: Phosphoinositide 3-kinase
POH: Progressive osseous heteroplasia
POLII: Polymerase II
PROMs: Patient-reported outcome measures
pri-miRNA: Primary miRNA transcript
PTEN: Phosphate and tension homolog deleted on chromosome ten
RCT: Randomized controlled trial
RISC: RNA-induced silencing complex
ROM: Range of motion
RT: Radiotherapy
RUNX2: Runt-related transcription factor 2
SARS-CoV-2: Severe acute respiratory syndrome coronavirus 2
siRNA: Small interfering RNA
snoRNA: Small nucleolar RNA
snRNA: Small nuclear RNA
SOX9: SRY-box transcription factor 9
SP: Substance P
TBI: Traumatic brain injury
TGFβ: Transforming growth factor β
THA: Total hip arthroplasty
TLR: Toll-like receptor
TNFα: Tumor necrosis factor α
tRNAs: Transfer RNA
WBCT: Whole-body computed tomography
XPO5: Exportin 5
